# Sex differences in the association between Body Mass Index and cognitive function in Parkinson disease: a cross-sectional study

**DOI:** 10.3389/fnut.2024.1420225

**Published:** 2024-07-05

**Authors:** QiuShuang Wang, Jing Bian, Yi Sun, YaoZhou Shi, ZiXuan Zhao, HuaShuo Zhao

**Affiliations:** ^1^Department of Biostatistics, School of Public Health, Xuzhou Medical University, Xuzhou, Jiangsu, China; ^2^The Affiliated Hospital of Xuzhou Medical University, Xuzhou, Jiangsu, China; ^3^Department of Public Administration, School of Health Economics and Management, Nanjing University of Chinese Medicine, Nanjing, China

**Keywords:** Parkinson’s disease, Body Mass Index, cognitive function, sex differences, logistic regression

## Abstract

**Objective:**

This study utilized a binary logistic regression model to explore the relationship between Body Mass Index (BMI) and cognitive function in Parkinson’s disease (PD) patients.

**Methods:**

In this cross-sectional study, data were obtained from 1,005 Parkinson’s patients enrolled in the Parkinson’s Progression Markers Initiative (PPMI) from 2010 to 2023, including 378 females and 627 males. Cognitive function was assessed using the Montreal Cognitive Assessment (MoCA) scale, and the correlation between BMI and cognitive function was determined using binary logistic regression.

**Results:**

The median age of enrollment was 63.6 (56.2, 69.6) years old, including 378 (37.6%) females and 627 (62.4%) males. In the final adjusted model, a significant positive correlation was found between BMI and the prevalence of cognitive impairment in females (OR = 1.06, 95% CI = 1.01 ~ 1.12, *p* = 0.022), while no correlation was found in males (OR = 1.03, 95% CI = 0.99 ~ 1.08, *p* = 0.165). The results after categorizing BMI indicate that, among females, the risk of cognitive impairment increases for both groups with BMI ≥ 30 kg/m^2^ and those with 25 ≤ BMI < 30 kg/m^2^ compared to the reference group with BMI < 25 kg/m^2^, with a *p* for trend <0.001 indicating a stable and strong association between BMI and cognitive impairment in females. In males, the results were not significant. The trend of linear fitting was consistent with the above results.

**Conclusion:**

In female Parkinson’s patients, there is a positive correlation between BMI and cognitive impairment, while no correlation was found in male patients. This study provides new evidence of sex differences in the correlation between BMI and cognitive impairment among Parkinson’s patients. The role of sex differences in the relationship between BMI and cognitive impairment should be considered in future research.

## 1 Introduction

Parkinson’s disease (PD) is the second most common neurodegenerative disorder globally, affecting over 6 million people (1). The World Health Organization predicts that by 2030, there will be approximately 8.67 million people with PD worldwide (2). The primary features of PD include bradykinesia, rigidity, tremors, and impaired balance. The non-motor symptoms of PD appear many years earlier than the motor symptoms (3) and can even have a greater impact on patients than the motor symptoms. Non-motor symptoms include depression, anxiety, cognitive impairment, dementia, sleep disorders, and autonomic symptoms (4–6). Cognitive impairment is one of the common non-motor symptoms in PD patients, with considerable variation in progression, symptoms, and severity from the onset to the latest stages of the disease (7). Between 20 to 30% of patients exhibit mild cognitive impairment (8). Cognitive impairments in PD can affect the quality of life of patients and increase the burden on their families and caregivers. There are notable sex differences in the epidemiology and clinical features of PD (9, 10), and cognitive impairments also show sex-specific patterns. Studies indicate that male PD generally have poorer cognitive abilities, and in the severe stages of the disease, cognitive impairments progress more rapidly in males (11, 12). In males, deficits in attention, memory, verbal fluency, and facial emotion recognition are more common, while females tend to show poorer performance in visuospatial functions (13).

Obesity is defined as the abnormal or excessive expansion of white adipose tissue and has reached epidemic proportions, being considered a significant health issue (14). Obesity is associated with numerous diseases, including cardiovascular diseases (15), diabetes (16), and various cancers (17, 18). It is also a risk factor for PD (19) as adipose tissue-produced adipokines upregulate systemic inflammation and induce insulin resistance, thereby accelerating the disease progression (20, 21). Currently, the impact of obesity on cognitive impairment can be seen in two ways. The first is that obesity may lead to a decline in cognitive abilities (22, 23). Batsis et al. (24) and Xu et al. (25) found that obesity is associated with impaired cognitive function in older adults, dementia, Alzheimer’s disease, and vascular dementia, and that obesity increases the risk of developing these conditions. The second perspective could overturn our previous understanding, the “obesity paradox,” as some studies suggest that obesity may be a protective factor against cognitive impairment (26, 27). Ely et al. (28) explored the relationship between cognitive function and obesity in patients with heart failure. The study indicated that non-obese patients had a higher risk of cognitive impairment than obese patients. Qizilbash et al. (29) conducted a follow-up study to show that individuals with a lower body weight (BMI < 20 kg/m2) had a 34% higher risk of dementia compared to those with a healthy weight (95%CI = 29 ~ 38). The existence of the obesity paradox may include factors such as diet, exercise, genetics, and others, though the specific mechanisms remain unclear. Studies have found that there are sex differences in obesity, BMI, and levels of brain-derived neurotrophic factor (30). Lentoor et al. (31) studied a group of women in South Africa to explore the relationship between BMI and neurocognitive function scores in adult women, finding that women with higher BMI had significantly lower the Montreal Cognitive Assessment (MoCA) scores compared to women with lower BMI.

Previous research has suggested that obesity might lead to a decline in cognitive abilities, yet it might also act as a protective factor for cognitive function. Understanding the correlation between BMI and cognitive function among male and female Parkinson’s patients could provide a basis for preventing cognitive impairments in these patients. Therefore, we use data from the Parkinson’s Progression Markers Initiative (PPMI) database to explore the relationship between BMI and cognitive function and their sex differences. Understanding these sex differences can help provide more targeted prevention and treatment recommendations for different groups, assist clinicians in determining weight control targets for Parkinson’s patients, and support efforts to improve their cognitive functions.

## 2 Methods

### 2.1 Data sources

**Figure 1:**
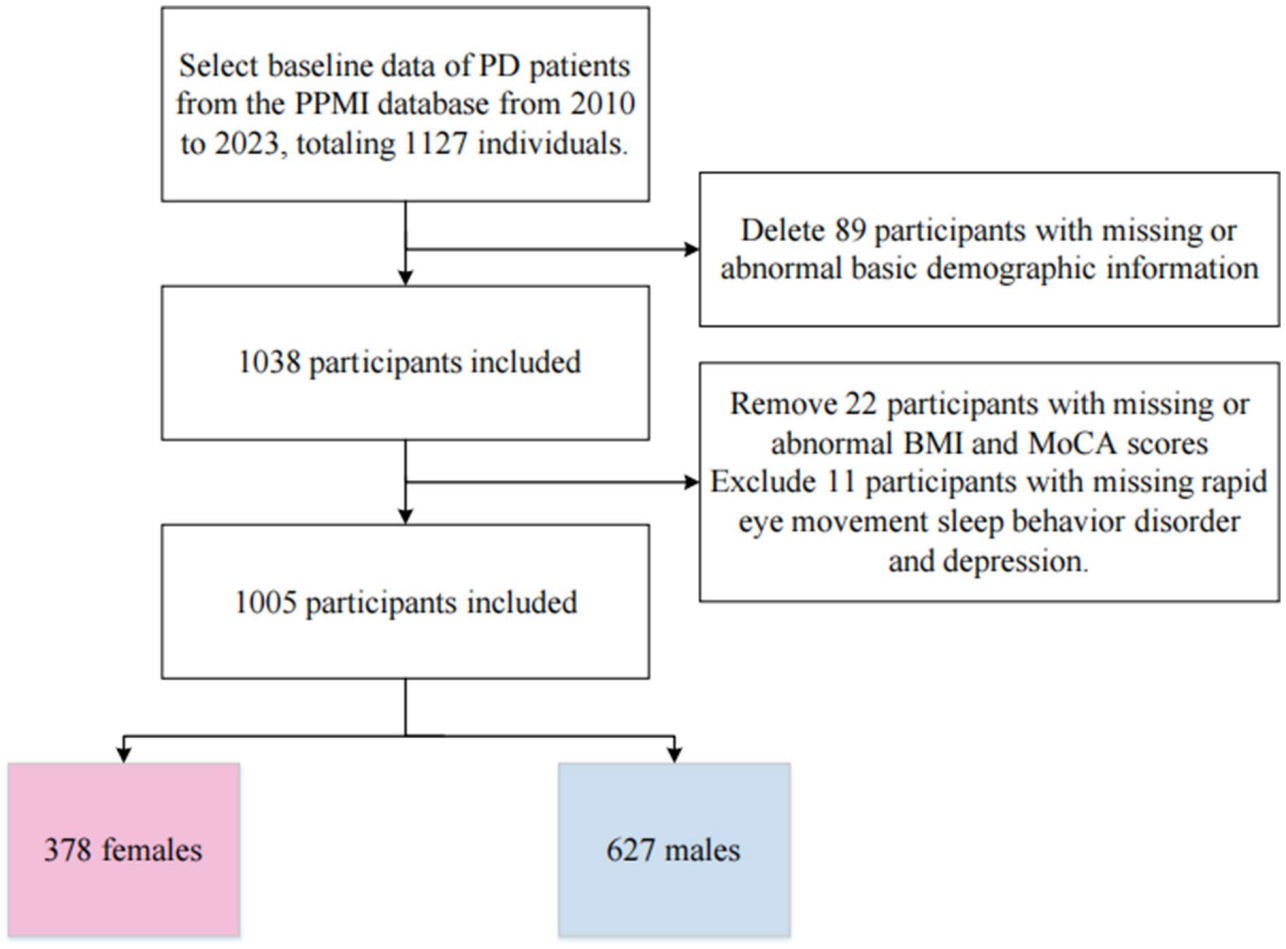
Participant inclusion flowchart.

We obtained data from the Parkinson’s Progression Markers Initiative (PPMI), a publicly available database. In 2010, The Michael J. Fox Foundation, along with a core group of academic scientists and industry partners, launched the PPMI to identify much-needed biomarkers for the onset and progression of Parkinson’s disease. Data used in the preparation of this article were obtained from the Parkinson’s Progression Markers Initiative (PPMI) database, RRID:SCR_006431.^1^ For up-to-date information on the study, visit www.ppmi-info.org. We included 1,127 Parkinson’s patients enrolled from 2010 to 2023, obtaining their baseline data. We excluded data for 89 participants due to missing baseline demographic information, 22 participants due to missing BMI and MoCA scores, and 11 participants due to missing other scale data. Ultimately, 1,005 participants were included in the study, consisting of 378 females and 627 males. The flowchart of participant inclusion and exclusion is shown in Figure 1. None of our participants received treatment at baseline, but underwent confirmative assessments, including clinical and cognitive evaluations, imaging examinations, and biological sampling, which were approved by the local participant Central Institutional Review Board. All participants provided written informed consent prior to enrollment.

### 2.2 Measurement and definition of variables

#### 2.2.1 Demographic and clinical variables

The baseline data included basic demographic and clinical variables, documenting race, age, years of education, family history, and Hoehn-Yahr stage, as well as disease duration. Additionally, all participants completed the Rapid-Eye-Movement Sleep Behavior Disorder Screening Questionnaire (32) and the 15-items Geriatric Depression Scale (33) as measures of sleep disorders and depression. The scores of the Movement Disorder Society Unified Parkinson’s Rating Scale part III to illustrate motor symptom severity.

#### 2.2.2 Assessment of BMI

Body Mass Index (BMI) is the most commonly used indicator to assess overweight and obesity (34). In this study, the participants’ heights were measured with a ruler while they were barefoot. Their weights were measured in kilograms (kg) and recorded to one decimal place. Body Mass Index (BMI) is defined as weight (kg) divided by the square of height (m). For adults, BMI categories are defined as: underweight, BMI < 18.5 kg/m^2^; normal weight, 18.5 ≤ BMI < 25 kg/m^2^; overweight, 25 ≤ BMI < 30 kg/m^2^; obese, BMI ≥ 30 kg/m^2^. Since our sample included only 14 individuals with a BMI < 18.5 kg/m^2^, we combined them into the 18.5 ≤ BMI < 25 kg/m^2^ category. Thus, our BMI categories are as follows: under−/normal weight, BMI < 25 kg/m^2^; overweight, 25 ≤ BMI < 30 kg/m^2^; and obese, BMI ≥ 30 kg/m^2^.

#### 2.2.3 Assessment of cognitive function

The Montreal Cognitive Assessment (MoCA) is used to evaluate neurocognitive function (35). The MoCA test includes multiple cognitive tests, covering areas such as memory, executive functions, language, attention, calculation ability, visual–spatial skills, and abstract thinking. The total score is 30 points. Therefore, MoCA scores range from 0 to 30, with higher scores indicating better neurocognitive function. MoCA score of 26 or higher indicates no cognitive impairment, while a score below 26 (MoCA <26) can be defined as mild cognitive impairment (36). The use of the MoCA scale is widely adopted in clinical practice, particularly for screening patients who may have cognitive issues.

### 2.3 Statistical analysis

Continuous variables are presented as mean (standard deviation, SD) or median (P25, P75), while categorical variables are presented as frequencies and percentages (*n*, %). The analysis of categorical variables uses the Chi-square test, and sex differences in descriptive variables are tested using the Student t-test or Mann–Whitney U test. Initially, univariate logistic regression is performed separately for male and female to determine if there is a relationship between each independent variable and the dependent variable, to understand the impact of each factor on the dependent variable in both groups. Next, multicollinearity is tested using the Variance Inflation Factor (VIF) method, with a VIF value of 5 or greater indicating multicollinearity. Subsequently, multivariable logistic regression models are constructed separately for females and males, using binary logistic regression to calculate odds ratio (OR) and 95% confidence interval (CI), to evaluate the correlation between BMI and cognitive impairment. Three models were constructed in total: Model 1: crude model without any covariate adjustment; Model 2: adjusted only for age and race; Model 3: builds on Model 2 by adding disease duration, Years of education, family history, and Hoehn-Yahr stage. Finally, to more accurately describe complex relationships within the data, and to visually demonstrate trends and features of the data, aiding in a deeper understanding of the relationships between data, linear fitting was performed. All analyses were conducted using SPSS version 25.0 and R (version 4.2.3). The test level is set at a *p*-value of 0.05.

## 3 Results

### 3.1 Characteristics of the participants

A total of 1,005 Parkinson’s patients were included, with a median age of enrollment of 63.6 (56.2, 69.6) years old. Of these, 378 (37.6%) were female and 627 (62.4%) were male. Among the females, 95 (25.1%) suffered from cognitive impairment, and among the males, 182 (29.0%) suffered from cognitive impairment. The characteristics of participants are summarized by sex in Table 1. The median age of females was 62.7 (55.4, 70.1) years old and that of males was 64.0 (56.7, 69.4) years old. There was a significant difference in the years of education between males and females (*p* = 0.001), with a higher proportion of males having a higher education level than females (85.8% vs. 77.8%). The study population was primarily White people (93%). The median duration of disease was 0.6 (0.3, 1.5) years, with Hoehn-Yahr stages between 1 and 3, without any more severe stages. The median disease duration for females was 0.7 (0.3, 1.9) years, and for males, it was 0.6 (0.3, 1.3) years, with a statistically significant difference between the two. The BMI for females was 24.9 (22.1, 28.9) and the MoCA score was 28.0 (25.0, 29.0), compared to the males’ BMI of 26.7 (24.4, 29.6) and MoCA score of 27.0 (25.0, 29.0), both showing significant statistical differences (*p* < 0.05). There were no statistically significant differences in sleep disturbances, depression, and motor symptom severity between the two groups.

**Table 1:** Baseline characteristics of the study population by sex.

Variables	Total	Female	Male	*Z*/*χ*2	*p*
	1,005	378	627		
Age (years old), median (P25,P75)	63.6 (56.2, 69.6)	62.7 (55.4, 70.1)	64.0 (56.7, 69.4)	−0.513	0.608
Years of education, *n* (%)					
<13 years	173 (17.2)	84 (22.2)	89 (14.2)	10.664	0.001
≥13 years	832 (82.8)	294 (77.8)	538 (85.8)		
Race, *n* (%)					
White	935 (93.0)	348 (92.1)	587 (93.6)	0.882	0.348
Non-white	70 (7.0)	30 (7.9)	40 (6.4)		
Family history, *n* (%)					
Yes	349 (34.7)	137 (36.2)	212 (33.8)	0.615	0.433
No	656 (65.3)	241 (63.8)	415 (66.2)		
Hoehn-Yahr, *n* (%)					
Stage 1	338 (33.6)	116 (30.7)	222 (35.4)	13.564	0.001
Stage 2	647 (64.4)	247 (65.3)	400 (63.8)		
Stage 3	20 (2.0)	15 (4.0)	5 (0.8)		
Disease duration (years), median (P25, P75)	0.6 (0.3, 1.5)	0.7 (0.3, 1.9)	0.6 (0.3, 1.3)	−2.889	0.004
BMI (kg/m2), median (P25, P75)	26.2 (23.9, 29.4)	24.9 (22.1, 28.9)	26.7 (24.4, 29.6)	−5.908	<0.001
Moca, median (P25, P75)	27.0 (25.0, 29.0)	28.0 (25.0, 29.0)	27.0 (25.0, 29.0)	−2.582	0.010
RBDSQ, median (P25, P75)	3.0 (2.0, 6.0)	3.0 (2.0, 5.0)	3.0 (2.0, 6.0)	1.894	0.058
GDS-15, median (P25, P75)	2.0 (1.0, 3.0)	2.0 (0.8, 4.0)	2.0 (1.0, 3.0)	−0.768	0.443
MDS-UPDRS3, median (P25, P75)	21.0 (15.0, 29.0)	20.5 (14.0, 29.0)	21.0 (15.0, 29.0)	0.576	0.565

Continuous variables are expressed as mean (SD) or median (P25, P75), and categorical variables are expressed as n (%). RBDSQ, Rapid-Eye-Movement Sleep Behavior Disorder Screening Questionnaire; GDS-15, the Geriatric Depression Scale-15 items; MDS-UPDRS3, the Movement Disorder Society Unified Parkinson’s Rating Scale part III.

**Figure 2:**
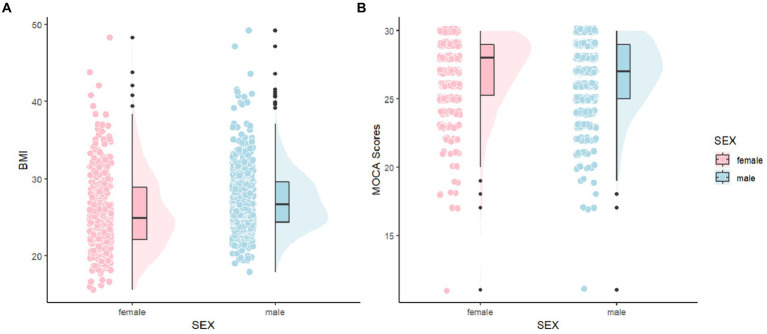
Comparisons of MoCA and BMI between female and male. MoCA, The Montreal Cognitive Assessment and BMI, Body Mass Index.

To provide a more intuitive comparison of the distribution differences in MoCA scores and BMI between males and females, as well as to better understand the variations and characteristics among individuals, we created Figure 2. In Figure 2A, it is observable that the distribution area for males is larger in higher BMI ranges and the mean is also higher compared to females, indicating a greater number of males with higher BMI. In Figure 2B, the distribution of MoCA scores for both males and females is primarily concentrated between 26 and 30 points, suggesting that most Parkinson’s patients do not exhibit cognitive impairments, likely because our sample mainly consists of relatively recent cases with about a year of disease progression. For females, the distribution in the MoCA range of 25–30 points is skewed, whereas it is normal for males, with females tending to cluster at higher MoCA scores and having a higher average score than males.

### 3.2 Univariate logistic regression analyses

To determine if there is a relationship between individual independent variables and the dependent variable, and to understand the impact of each factor on the dependent variable, we performed univariate logistic regression analyses. The results are shown in Table 2. BMI was associated with cognitive impairment in women (OR = 1.07; 95%CI = 1.02 ~ 1.12). However, there was no statistically significant association with cognitive impairment in men (OR = 1.02; 95%CI = 0.98 ~ 1.06). Age, years of education, race, and disease duration showed statistically significant associations with cognitive impairment either in men or women, whereas family history and Hoehn-Yahr stage showed no significant association with cognitive impairment.

**Table 2:** Univariate logistic regression analysis of cognitive impairment in females and males.

	Female		Male	
Variables	OR (95%CI)	*p-*value	OR (95%CI)	*p*-value
Age	1.05 (1.02 ~ 1.08)	<0.001	1.04 (1.02 ~ 1.06)	<0.001
Years of education				
<13 years	1 (Ref.)		1 (Ref.)	
≥13 years	0.36 (0.21 ~ 0.60)	<0.001	0.73 (0.45 ~ 1.17)	0.194
Race				
White	1 (Ref.)		1 (Ref.)	
Non-white	3.89 (1.82 ~ 8.32)	<0.001	2.11 (1.10 ~ 4.04)	0.024
Family history				
Yes	1 (Ref.)		1 (Ref.)	
No	0.64 (0.40 ~ 1.02)	0.063	0.80 (0.56 ~ 1.15)	0.230
Hoehn-Yahr				
Stage 1	1 (Ref.)		1 (Ref.)	
Stage 2, 3	1.33 (0.79 ~ 2.23)	0.286	1.39 (0.96 ~ 2.01)	0.083
Disease duration	1.07 (0.95 ~ 1.21)	0.247	1.16 (1.03 ~ 1.30)	0.018
BMI	1.07 (1.02 ~ 1.12)	0.004	1.02 (0.98 ~ 1.06)	0.355
RBDSQ	0.96 (0.88 ~ 1.05)	0.382	1.03 (0.98 ~ 1.09)	0.284
GDS-15	1.10 (1.02 ~ 1.19)	0.010	1.05 (0.98 ~ 1.11)	0.168
MDS-UPDRS3	1.02 (0.99,1.04)	0.141	1.03 (1.01,1.04)	0.003

RBDSQ, Rapid-Eye-Movement Sleep Behavior Disorder Screening Questionnaire; GDS-15, the Geriatric Depression Scale-15 items; MDS-UPDRS3, the Movement Disorder Society Unified Parkinson’s Rating Scale part III.

### 3.3 Multivariable logistic regression analyses

Combining univariate analysis of variance, we tested for multicollinearity using the Variance Inflation Factor (VIF) method. VIF directly reflects the correlation among independent variables. By comparing VIF values, we can visually assess the degree of multicollinearity, which helps to quickly identify independent variables that may have collinearity issues. VIF value of 5 or above indicates significant multicollinearity. As shown in Table 3, the VIF values for both the male and female groups were less than 5, indicating no multicollinearity among the independent variables in the model.

**Table 3:** The variance inflation factor (VIF) of variables in female and male.

	VIF	
Variables	Female	Male
Age	1.07	1.04
Disease duration	1.17	1.04
BMI	1.03	1.01
Years of education	1.08	1.02
Race	1.02	1.00
Family history	1.09	1.03
Hoehn-Yahr	1.11	1.05

Multivariate logistic regression analysis was used to evaluate the association between BMI and cognitive impairment among female and male PD patients separately. As shown in Table 4, in the female group, Models 1, 2, and 3 all demonstrated a significant positive correlation between BMI and cognitive impairment. In Model 3 (the fully adjusted model), the continuous variable BMI was significantly positively correlated with cognitive impairment (OR = 1.06, 95%CI = 1.01 ~ 1.12). After transforming the continuous variable into categorical variables, with BMI < 25 kg/m^2^ as the reference, the OR for the 25 ≤ BMI < 30 kg/m^2^ group was 1.83 (95%CI = 1.02 ~ 3.30, *p* = 0.043), and for the BMI ≥ 30 kg/m^2^ group, the OR was 3.34 (95%CI = 1.71 ~ 6.53, *p* < 0.001). This indicates that compared to the BMI < 25 kg/m^2^ group, the probability of cognitive impairment was 1.83 times higher in the 25 ≤ BMI < 30 kg/m^2^ group, and 3.34 times higher in the BMI ≥ 30 kg/m^2^ group. *p* for trend can be used to assess the stability of the model; it provides a means to evaluate the trend of a variable in the model and its impact on the dependent variable, thereby helping to judge the model’s rationality and stability. If *p* for trend is significant, it suggests that the relationship between the variable trend and the dependent variable in the model has certain stability and reliability. In Model 3, the OR values for overweight and obesity in PD patients were 1.83 (95%CI = 1.02 ~ 3.30) and 3.34 (95%CI = 1.71 ~ 6.53), respectively, compared to normal weight, with *p* for trend<0.001, indicating that in our model, the trend of BMI is stable in relation to cognitive impairment. Similarly, in Models 1 and 2, *p* for trend was also less than 0.001, similarly indicating a stable relationship between the trend of BMI and cognitive impairment.

**Table 4:** Association between BMI and cognitive impairment in females and males.

	Female			Male		
Variables	Model1	Model2	Model3	Model1	Model2	Model3
BMI	1.07 (1.02 ~ 1.12, 0.004)	1.07 (1.02 ~ 1.13, 0.005)	1.06 (1.01 ~ 1.12, 0.022)	1.02 (0.98 ~ 1.06, 0.355)	1.03 (0.98 ~ 1.07, 0.270)	1.03 (0.99 ~ 1.08, 0.165)
BMI categories						
BMI < 25 kg/m^2^	1 (Ref.)	1 (Ref.)	1 (Ref.)	1 (Ref.)	1 (Ref.)	1 (Ref.)
25 ≤ BMI < 30 kg/m^2^	2.31 (1.34 ~ 4.00, 0.003)	1.97 (1.12 ~ 3.49, 0.019)	1.83 (1.02 ~ 3.30, 0.043)	1.27 (0.85 ~ 1.90, 0.252)	1.29 (0.86 ~ 1.96, 0.221)	1.34 (0.88 ~ 2.04, 0.170)
BMI ≥ 30 kg/m^2^	3.30 (1.78 ~ 6.12, <0.001)	3.58 (1.88 ~ 6.80, <0.001)	3.34 (1.71 ~ 6.53, <0.001)	1.21 (0.74 ~ 1.99, 0.445)	1.24 (0.75 ~ 2.06, 0.396)	1.28 (0.77 ~ 2.14, 0.340)
*p* for trend	<0.001	<0.001	<0.001	0.433	0.430	0.374

Model 1: not adjusted.

Model 2: adjusted for age and race.

Model 3: adjusted for model 2 plus disease duration, years of education, family history and Hoehn-Yahr stage.

In the male group, Models 1, 2, and 3 showed no significant statistical association between BMI and cognitive impairment in PD patients (*p* > 0.05). Even after transforming the continuous variable into categorical variables, as BMI changed, there was still no significant statistical difference in its relationship with cognitive impairment (p > 0.05). The *p* for trend was also greater than 0.05, indicating no significant statistical relevance. These results suggest that in male PD patients, BMI may not have a direct relationship with the occurrence of cognitive impairment.

### 3.4 Linear fit of BMI and cognitive impairment

**Figure 3:**
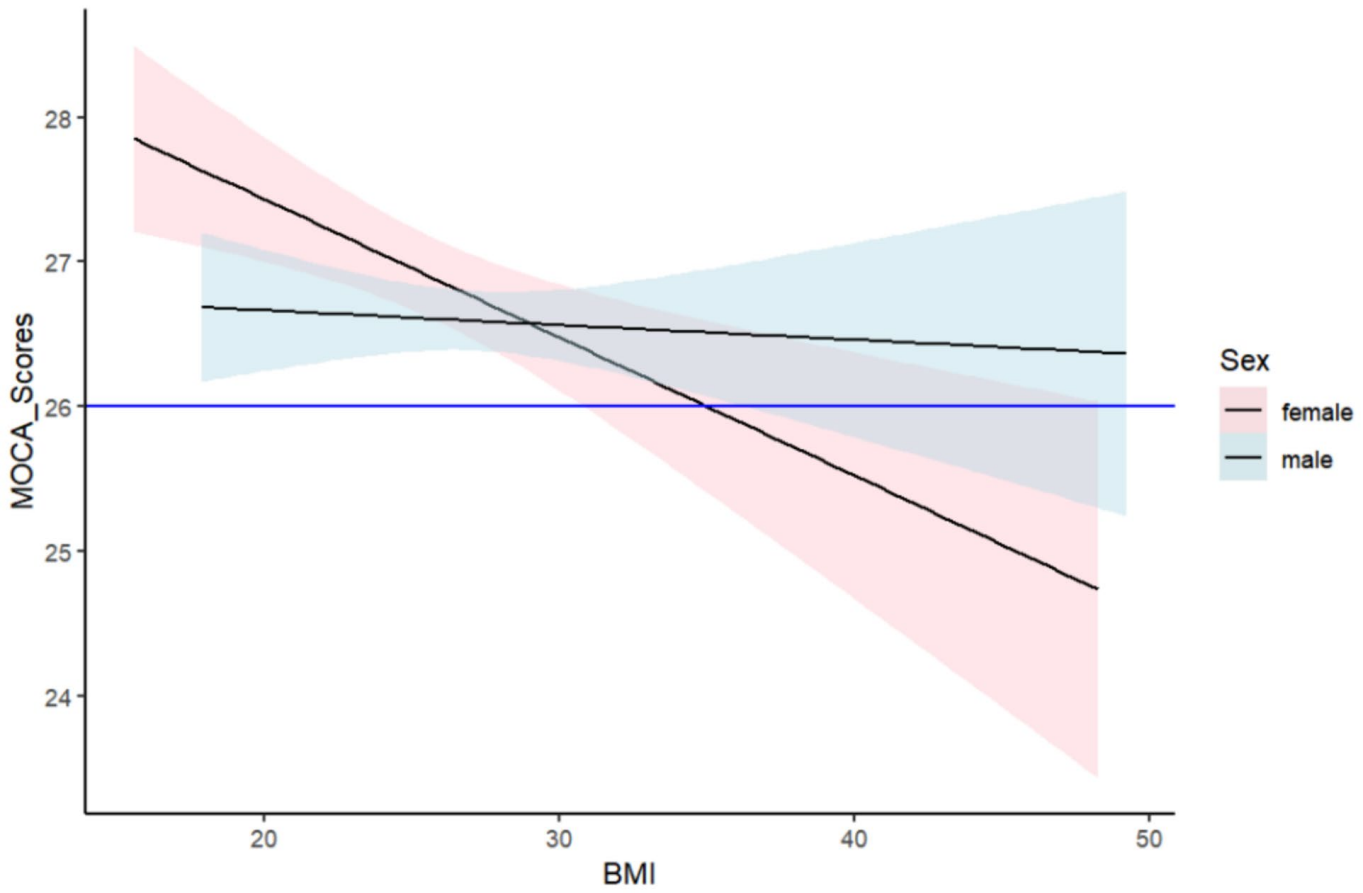
Linear fit graph of BMI and cognitive impairment.

We conducted linear tests on the two groups of data. In women, the P for nonlinearity was 0.32, and in men, it was 0.18, indicating no significant non-linear relationship in the data. In such cases, linear fitting is a preferable choice. Although linear fitting may not capture the complex relationships between variables in some instances, it has simplicity and wide applicability, which can help roughly observe the trends between two variables. In Figure 3, we can observe that in women, as BMI increases, MoCA scores gradually decrease, implying that in women, cognitive impairment becomes more severe with an increase in BMI, consistent with our previous research findings. In men, this change is not as pronounced as in women, but it can be observed that in men, as BMI increases, MoCA scores are also slowly decreasing.

## 4 Discussion

In this study, we found that BMI is positively correlated with the incidence of cognitive impairment in women, and this relationship remains stable after controlling for covariates. For every one-unit increase in BMI, the incidence rate of cognitive Impairment increases by 6%. Although the OR value of 1.06 seems small, the impact accumulates with gradual increases in BMI. For example, considering larger changes in BMI (such as increases of 5 or 10 units), the effect on the risk of cognitive impairment could be more significant. When BMI is segmented, the risk in women with BMI ≥ 30 kg/m^2^ is 234% higher compared to the reference BMI < 25 kg/m^2^. Further analysis with P for trend<0.001 indicates a stable and strong association between BMI and cognitive impairment in women. Our subsequent linear fitting also observed consistent results. In models 1, 2, and 3, there was no correlation between BMI and cognitive impairment in men, and the relationship between them in the linear fit graph also showed very minor changes. The National Institutes of Health in the United States emphasizes the importance of biological sex and considers it a critical variable in rigorous research. Epidemiological and clinical sex differences in PD are also quite common (37, 38). Analyzing the reasons for these results may be due to physiological differences between men and women, not just in body structure, but also including the distribution of fat and muscle, hormone levels, and changes in the body with age, such as muscle loss and fat gain; the mechanisms of PD are complex and also differ between sexes; the relationship between BMI and cognitive impairment is also very complex; the lifestyles and social relationships of men and women are different. Therefore, maintaining a healthy weight is crucial, not only to help us mitigate the impact of BMI on cognitive dysfunction but also vital for preventing other diseases. By maintaining a proper weight, we can appropriately increase the content of muscle tissue, which helps us resist various diseases. Although the association between BMI and cognitive function has not been shown in men, it is recommended that men also consistently maintain a healthy weight. Studying PD patients by sex can enhance the comprehensiveness and accuracy of research, making the results more fully reflect the actual situation of the population, and understanding sex differences can help provide more targeted and personalized prevention and treatment recommendations for different groups. Current methods for treating obesity primarily focus on lifestyle interventions, combinations of anti-obesity medications, endoscopy, and bariatric surgery (39). A healthier and more scientific approach is still to engage in physical exercise, which is not only cost-effective and efficient but also greatly helps improve Parkinson’s movement disorders (40).

Current research on the mechanisms linking obesity and cognitive decline primarily involves changes in brain structure and neural activity (41). Obesity can lead to changes in brain structure, such as reduced volumes of gray and white matter. These changes are associated with declines in cognitive functions, particularly in areas such as memory, executive functions, and attention; neurodegeneration, for instance, an increase in BMI is associated with reduced brain volume and gray matter atrophy (42); and oxidative stress (43), where increased levels of oxidative stress in obesity lead to an accumulation of reactive oxygen species. These reactive species can damage cell membranes, proteins, lipids, and nucleic acids, thus harming neurons and affecting their function and survival. Cognitive impairment is often associated with changes in brain structure (44, 45), such as reductions in gray and white matter volume, decreased cortical thickness, and enlarged ventricles. These changes can be detected through neuroimaging techniques like MRI. PET scans can detect the accumulation of beta-amyloid and tau proteins, which are associated with cognitive impairments like Alzheimer’s disease. The presence of these biomarkers is related to cognitive decline, and weight loss is also associated with these substances (46). Neuroimaging data provide a direct way to observe brain structure and function, offering insights into the relationship between BMI and cognitive impairment. Further research using neuroimaging techniques could explore these relationships and potentially identify intervention strategies to slow down or reverse cognitive decline caused by BMI. A deeper understanding of these relationships will help better predict and manage obesity-related cognitive impairments and provide a basis for clinical interventions. Additionally, factors such as lifestyle and metabolism can also influence the relationship between obesity and cognitive impairment. These mechanisms suggest that the relationship between obesity and cognitive impairment may result from the combined effects of multiple factors and pathways. Thus, preventing and treating obesity is not only critical for physical health but may also have significant implications for maintaining and enhancing cognitive functions. This also supports research into the relationship between BMI and neurocognitive functions.

In a previous study on the relationship between BMI and cognitive impairment among adult women in South Africa (31), it was found that women with higher BMI scores had significantly lower MoCA scores compared to those with lower BMI, across both global and domain-specific neurocognitive tasks of attention, memory, and executive function. This study did not employ logistic regression or more complex modeling, nor did it examine men, but the findings are consistent with our results regarding women. A group of researchers in China explored whether BMI is associated with cognitive function in Chinese patients with atrial fibrillation (47), finding that when BMI was below 24.56 kg/m2, each unit increase in BMI increased the cognitive function score by 0.43 points. Within the normal BMI range, the higher the BMI of patients with atrial fibrillation, the higher their cognitive function scores, suggesting that maintaining current weight is advisable for atrial fibrillation patients with a normal BMI. This particular trend may be due to the more complex mechanisms of cognitive decline in patients with atrial fibrillation, and the study did not perform a sex subgroup analysis; it was specific to patients with atrial fibrillation. Not only in specific groups but also studies in children and adolescents (48) show that obesity leads to impaired cognitive function. Research involving 3,323 children aged 6–16 aimed to identify the biological mediators between obesity and overweight and cognitive function in children and adolescents, establishing some biological links between obesity and cognitive function. Obesity has also been identified as a significant factor contributing to cognitive decline in the middle-aged population (24, 25). Although the obesity paradox exists, our study results lean toward the conclusion that “obesity increases the risk of cognitive impairment,” especially among women. Forbes et al. (49) also utilized 8-year longitudinal follow-up data from the PPMI database, establishing linear mixed-effects models to examine the association between baseline factors and changes in cognition, evaluated by the MoCA over time. They found that higher BMI was associated with a faster decline in MoCA scores, which is consistent with our findings. It is noteworthy that some studies (46) have found that patients who lose weight exhibit faster cognitive decline. We need to distinguish between these two measures as they provide different information. BMI is a value calculated based on a person’s weight and height and can serve as a standardized measure of health. Weight change refers to the actual increase or decrease in weight over a period. Weight loss is a dynamic process that directly reflects changes in weight, while BMI is a static indicator that assesses health.

Currently, there are few studies on the relationship between BMI and cognitive impairment in PD patients. This research included 1,005 PD patients, accounting for factors such as sex, race, years of education, family history, Hoehn-Yahr stage, and disease duration. We conducted univariate and multivariate logistic regression analyses as well as linear fitting for both males and females to explore the relationship between the two factors. Understanding sex differences helps provide more targeted prevention and treatment recommendations for different groups and assists clinicians in determining weight control targets for Parkinson’s patients, thereby providing a basis for improving cognitive functions in these patients. This study also has some limitations. Firstly, it is a cross-sectional study, so causal conclusions cannot be drawn. Additionally, the relationship between BMI and cognitive impairment may be influenced by confounding factors such as vascular comorbidities or diabetes. Secondly, BMI does not distinguish between body fat and lean mass, so using BMI as a variable has its limitations; in the future, more comprehensive indicators to reflect the degree of obesity could be used. Thirdly, our study primarily involved Caucasian participants, and results may differ for other races, which could limit the external applicability of our findings. Lastly, this study subjects are not monogenic PD cohorts (monogenic PD cohorts refer to a group of PD patients caused by known single gene mutations). In the future, we can study monogenic PD cohorts. By researching patients carrying specific gene mutations, we can gain a deeper understanding of the pathological mechanisms of PD. This can help reveal the molecular and cellular basis of disease progression.

## 5 Conclusion

Based on an analysis of baseline data from PPMI, this study employed univariate logistic regression, multivariate logistic regression, and linear fitting to elucidate the relationship between BMI and cognitive function in PD patients, separately for males and females. The results indicate a positive correlation between BMI and cognitive impairment in female Parkinson’s patients, whereas no such correlation was found in male Parkinson’s patients. Therefore, it is crucial for female Parkinson’s patients to maintain a normal weight. Although there is no correlation between BMI and cognitive impairment in male Parkinson’s patients, maintaining a healthy weight is still necessary. This study provides new evidence on the sex differences in the correlation between BMI and cognitive impairment in PD patients. The role of sex differences in the relationship between BMI and cognitive function should be considered in future research.

## Data availability statement

The original contributions presented in the study are included in the article/Supplementary material, further inquiries can be directed to the corresponding authors.

## Author contributions

QW: Conceptualization, Software, Visualization, Writing – original draft. JB: Investigation, Methodology, Writing – review & editing. YSu: Data curation, Formal analysis, Writing – review & editing. YSh: Data curation, Investigation, Resources, Writing – review & editing. ZZ: Conceptualization, Project administration, Supervision, Writing – review & editing. HZ: Conceptualization, Data curation, Resources, Software, Writing – review & editing.

## Footnotes

^1^ www.ppmi-info.org/access-data-specimens/download-data
